# Identification and Functional Exploration of the *ALKBH* Gene Family in Oriental Melon Fruit Ripening

**DOI:** 10.3390/ijms26094254

**Published:** 2025-04-29

**Authors:** Chong Zhang, Xinqi Guo, Ying Zhang, Hongbo Pang, Qiang Chen

**Affiliations:** 1 Department of Life Science, Shenyang Normal University, Shenyang 110034, China; nancy220429@126.com (C.Z.); a13700189259@126.com (X.G.); f5944@163.com (Y.Z.); panghongbo800206@163.com (H.P.); 2 Experimental Teaching Center, Shenyang Normal University, Shenyang 110034, China

**Keywords:** *Cucumis melo*, RNA methylation, ALKBH, fruit ripening, ethylene synthesis

## Abstract

N6-methyladenosine (m^6^A) methylation functions as a vital post-transcriptional and epigenetic modification in higher plants regulated by α-ketoglutarate-dependent dioxygenases (ALKBH). However, the role of ALKBH genes in oriental melon (*Cucumis melo* L.) fruit ripening has not been explored. Therefore, we treated oriental melon with an exogenous m^6^A demethylase inhibitor (mechlorfenamic acid) then analyzed endogenous ethylene production and ripening-related indicators to explore the effects of m^6^A methylation on ripening. Bioinformatics and real-time quantitative PCR analyses were used to determine the impact of *ALKBH* genes on key ethylene synthesis gene expression. Treatment effectively inhibited endogenous ethylene production, firmness changes, and soluble solid contents, thereby extending fruit ripening. Eight *ALKBH* gene family members belonging to five major groups were identified in the melon genome. All members were expressed in ripening fruits, with different expression patterns during ripening. *CmALKBH6*, *CmALKBH7*, and *CmALKBH8* expression was inhibited by an ethylene inhibitor (1-methylcyclopropene). The transient overexpression (OE) of *CmALKBH8* in oriental melon led to the increased expression of the ethylene synthesis genes *CmACS1*, *CmACS2*, and *CmACO1*. In summary, the ethylene-regulated gene *CmALKBH8* may participate in oriental melon fruit ripening regulation by modulating the methylation levels of ethylene synthesis-related genes. These findings help us better understand how m^6^A methylation regulates melon ripening.

## 1. Introduction

RNA modification describes post-transcriptional nucleic acid modification that regulates gene expression without altering DNA sequences and can be inherited through cell mitosis and meiosis [1,2]. In higher plants, N6-methyladenosine (m^6^A) methylation is the most abundant widely occurring form of RNA modification, occurring at the N-6 position of adenosine. m^6^A methylation is a dynamic and reversible modification involved in regulating gene expression, RNA splicing, localization, RNA stability, mRNA degradation, and circular RNA translation [2,3]. The rapid development of sequencing technologies has resulted in the m^6^A methylation maps of various physiological processes in different plants, such as in *Arabidopsis* [4], rice [5], maize [6], cabbage [7], and apple [8]. These maps help us better understand the regulatory systems of m^6^A methylation throughout plant development from growth to maturation and senescence and under stress conditions. Research on m^6^A methylation in fruit ripening has also benefited from technological advances, particularly the application of MERIP-seq technology to fruits such as tomato, kiwifruit, and strawberry, which has revealed distinct patterns of m^6^A regulation in climacteric and non-climacteric fruits [9,10,11]. In climacteric fruits like tomato and kiwifruit, significant m^6^A modifications occur mainly at stop codons and 3′UTRs, with m^6^A levels gradually decreasing during ripening [9,10]. The m^6^A levels tend to increase during the ripening of non-climacteric fruits like strawberry, which may enhance the translation efficiency of genes involved in ABA biosynthetic signaling, thus speeding up the ripening process [11].

In higher plants, the m^6^A demethylase ALKBH functions as a member of the 2-oxoglutarate (2OG) and Fe(II)-dependent dioxygenase superfamily [12,13]. Researchers have identified all ALKBH family members through genome-wide studies across various plants, such as *Arabidopsis* [12], tomato [13], potato [14], and citrus [15]. *ALKBH* genes in different plants function to control flowering [16] and responses to abiotic stress [17,18,19,20,21], while also influencing fruit ripening. The m^6^A demethylase *SlALKBH2* can bind to the transcript of the DNA demethylase gene *SlDML2* to regulate m^6^A demethylation during tomato fruit ripening in plants. The mutation of *SlALKBH2* results in elevated m^6^A levels together with reduced *SlDML2* mRNA abundance, delaying fruit ripening [22]. The thioredoxin reductase SlNTRC interacts with *SlALKBH2* and catalyzes its reduction, thereby regulating m^6^A levels and fruit ripening [23,24]. In kiwifruit, *AcALKBH10* governs m^6^A methylation in ripening-related genes, which impacts soluble sugar and organic acid levels, thus affecting the ripening process and the final fruit quality [9]. In strawberry, *FvALKBH10B* mutation delays ripening and causes hypermethylation of 1859 genes, revealing the role of the *FvABF3-FvALKBH10B*-*FvSEP3* module in controlling fruit ripening [25].

The ripening of climacteric oriental melon (*Cucumis melo* L.) fruits mainly depends on ethylene regulation. Fruit softening, color formation, sugar accumulation, and aroma synthesis depend heavily on ethylene biosynthesis and signaling [26,27,28,29]. Post-harvest tomato fruit cold storage analysis through Rm^6^A-seq and RNA-seq unveiled that the key ethylene synthesis genes, ethylene response factor 2 (ERF2), 1-aminocyclopropane-1-carboxylate oxidase (ACO), and 1-aminocyclopropane-1-carboxylate synthase (ACS), were negatively correlated with overall m^6^A levels [30]. The m6A writer inhibitor 3-deazaadenosine and the m^6^A eraser inhibitor meclofenamic acid (MA) when directly injected into immature tomato fruits either sped up or slowed down the ripening process while affecting the expression levels of key ethylene synthesis genes *SlACS4* and *SlACO3* [31]. These results imply that m^6^A methylation functions to manage ethylene synthesis for regulating tomato fruit ripening. However, the role of m^6^A methylation in ethylene-regulated melon fruit ripening and the potential feedback regulation mechanism have not been reported.

To understand the impact of m^6^A methylation on oriental melon fruit ripening, we treat oriental melon fruits with exogenous demethylase inhibitors then analyze endogenous ethylene production and ripening-related indicators to explore the effects of m^6^A methylation on ripening. The identification of *ALKBH* genes within the melon genome is achieved through the use of bioinformatics tools, and real-time quantitative PCR (qPCR) is employed to screen members exhibiting significant expression during ripening. The overexpression (OE) of candidate *ALKBH* genes is used to investigate their impact on the expression of key ethylene synthesis genes. This study sheds light on *ALKBH*’s contributions to climacteric fruit ripening and elucidates the feedback regulation mechanism between ethylene and m^6^A methylation in oriental melon fruit ripening.

## 2. Results

### 2.1. Effects of Direct MA Injection on Oriental Melon Fruit Ripening

**Figure 1:**
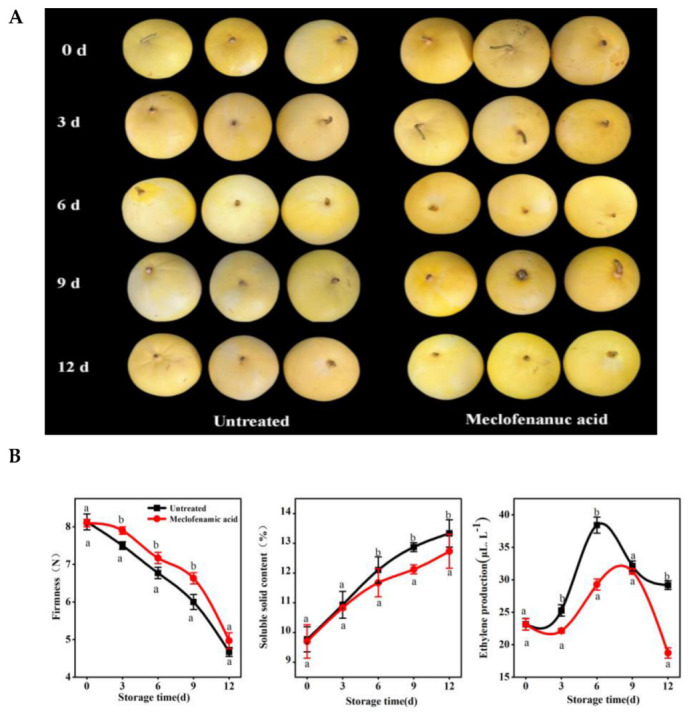
Effects of direct injection of meclofenamic acid on oriental melon fruit ripening. (**A**) Phenotypes of the oriental melon fruits after injection of MA. (**B**) The firmness, soluble solid content, and ethylene production of the oriental melon fruits were measured after injection. Data are represented as means ± standard deviation of three biological replicates (*n* = 6), and different lowercase letter indicate significant differences (Student’s test, *p* < 0.05).

MA was first reported as an m^6^A demethylase inhibitor in animals; more recent studies have shown that direct MA injection into tomato, a horticultural model crop, effectively alters fruit development. We studied the regulation of m^6^A methylation in fruit ripening by injecting MA into oriental melon fruits 25 days after pollination (at approximately 80% ripeness), then observed fruit phenotypes and measured ripening-related physiological indicators like endogenous ethylene production, firmness, and soluble solids content. Figure 1A shows that MA significantly altered fruit appearance, effectively delaying changes in peel color during ripening. Figure 1B shows that fruit firmness gradually decreased during ripening, with MA-treated fruits being significantly harder than the controls 3–12 days post injection. The soluble solids content increased during ripening, but MA treatment effectively delayed this increase six days post injection. MA also delayed the peak of ethylene release. Therefore, MA treatment could effectively delay oriental melon fruit ripening.

### 2.2. Identification of ALKBH Genes in the Melon Genome

A search for ALKBH genes within the melon genome relied on the ALKBH domain hidden Markov model (HMM) model downloaded from Pfam, along with *Arabidopsis* ALKBH protein sequences as queries for HMMER and BLASTP identification, validated by the CDD online website. Eight CmALKBH family members were identified (Table 1), which were unevenly distributed on melon chromosomes, with four members on chromosome 8. All proteins contained the 2OG-FeII-Oxy domain (Table S1). CmALKBH proteins contained 244 to 502 amino acids, resulting in molecular weights between 28.06 and 55.85 kDa and theoretical isoelectric points between 4.68 and 9.32. Subcellular localization prediction indicated that most CmALKBH genes (4/8) were located in the nucleus, two were in chloroplasts, and the remaining two were in the cytoplasm and extracellular space.

### 2.3. Phylogenetic Tree of ALKBH Proteins

**Figure 2:**
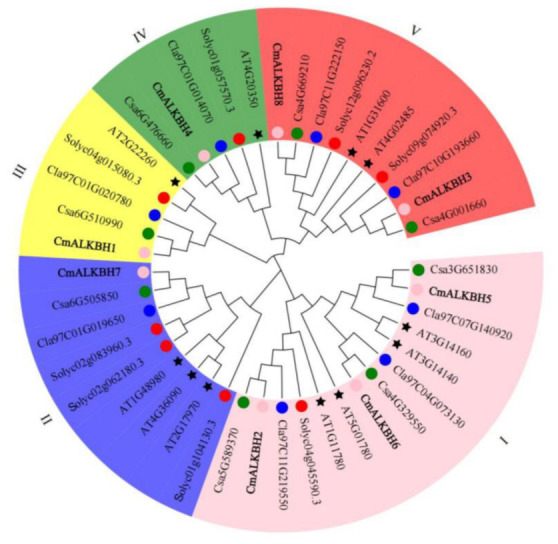
The neighbor-joining (NJ) phylogenetic tree of ALKBH proteins from *Arabidopsis thaliana*, *Solanum lycopersicum*, *Cucumis melo*, *Cucumis sativu*, and *Citrullus lanatus*. Black stars represent *Arabidopsis thaliana*, the red, green, pink, blue circles represent *Cucumis melo*, *Cucumis sativu*, and *Citrullus lanatus*.

To assess the evolutionary relationships of CmALKBH, protein sequences encoded by ALKBH genes from *Arabidopsis*, tomato, cucumber, watermelon, and melon were analyzed (Table S1; Figure 2). The 43 ALKBH genes from these species were divided into five subfamilies (I–V). Subfamily I had the most members in melon (three), followed by subfamily VI (two) and then the other subfamilies (one each). All subfamilies contained the same number of ALKBH proteins from melon, cucumber, and watermelon, indicating a closer evolutionary relationship among cucurbit species than with *Arabidopsis* and tomato.

### 2.4. Motifs and Gene Structures of ALKBH from Cucumis melo

**Figure 3:**
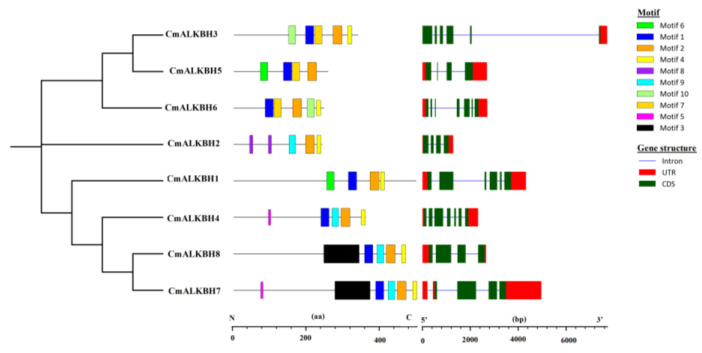
Distribution of conserved motifs and exon/intron structures in the predicted CmALKBH protein. Each motif is represented by a colored box. The length of box corresponds to the motif length. Red boxes represent exon, and green/blue lines represent upstream/downstream sequences.

We analyzed and compared the conserved motifs as well as gene structures of CmALKBH (Figure 3) and identified ten conserved motifs (Motif_1 to Motif_10) (Table S2). CmALKBH proteins shared similar motif compositions, with eight proteins containing Motif_2 and Motif_4, suggesting that these motifs play key roles. Most ALKBH proteins shared two common motifs, but different members had specific motif combinations. For example, all members except CmALKBH2 contained Motif_1, but only CmALKBH2 contained Motif_8, indicating a unique function. Motif_5 was found only in CmALKBH4 and CmALKBH7. Conservation within subfamilies was not high, indicating incomplete consistency with evolutionary relationships. *CmALKBH* genes showed different exon positions, with most genes having conserved exon numbers. *CmALKBH1*, *CmALKBH3*, *CmALKBH5*, and *CmALKBH6* had the fewest exons (four), whereas *CmALKBH2* and *CmALKBH4* had the most (seven).

### 2.5. Synteny Analysis of Melon ALKBH Genes

**Figure 4:**
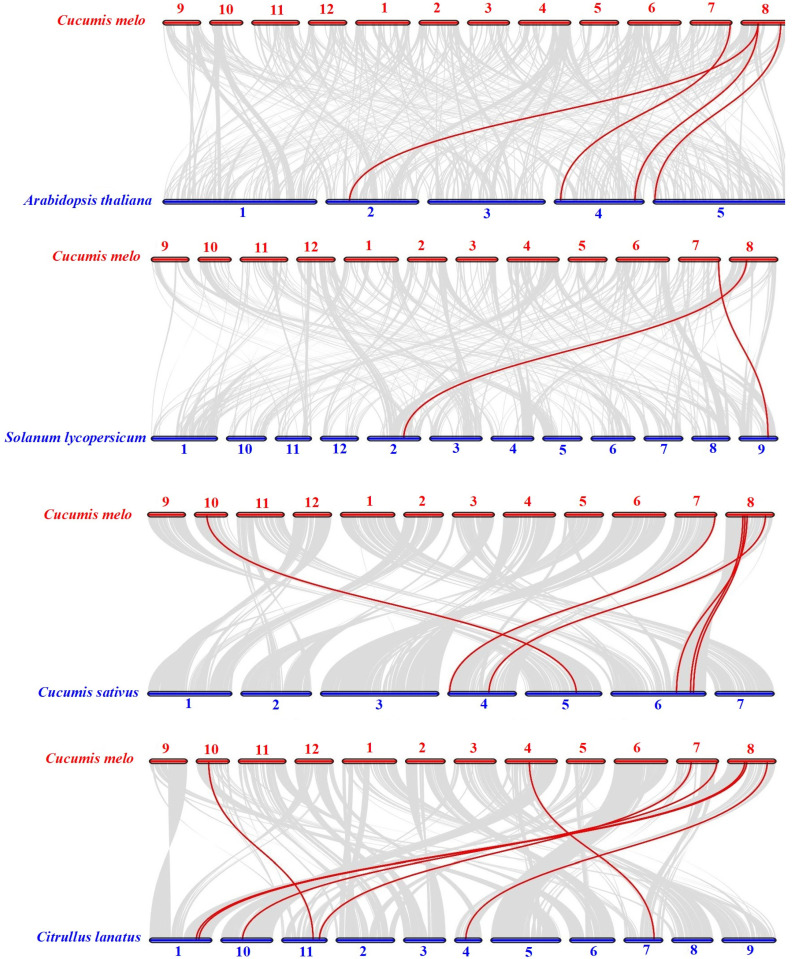
Synteny analysis of ALKBH genes in *Cucumis melo* (Cme), *Arabidopsis thaliana* (At), *Solanum lycopersicum* (Sl), *Cucumis sativus* (Csa), and *Citrullus lanatus* (Cla). Red lines display the collinear ALKBH genes among four pairs of plant genomes. The light gray lines denote collinear blocks.

We performed intra-genomic synteny analysis of eight *ALKBH* genes to study their expansion mechanisms. No tandem or segmental duplication events were found. Further analysis of synteny between melon and related species revealed syntenic relationships with watermelon, cucumber, *Arabidopsis*, and tomato for seven, six, four, and two melon *ALKBH* genes, respectively. The strongest synteny was observed between melon and watermelon/cucumber, suggesting recent divergence and high functional similarity (Figure 4).

### 2.6. Cis-Acting Elements in the Promoter Regions of Melon ALKBH Genes

**Figure 5:**
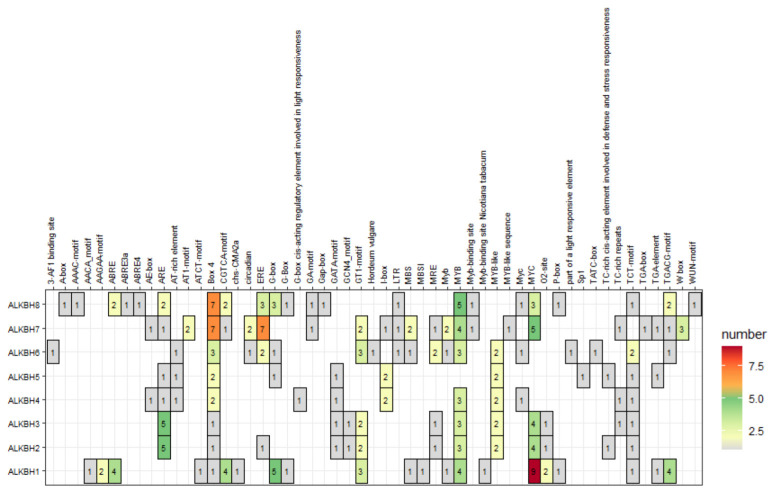
The statistics of cis-regulatory elements of each melon CmALKBH regulatory gene. The number represents the number of responsive elements in the promoter.

PlantCARE served to analyze cis-acting elements within the promoter regions for inferring the biological functions of *CmALKBH* genes. Most CmALKBH genes contain light-responsive elements, indicating potential light regulation. Hormone-responsive elements, including those for ethylene, abscisic acid, gibberellin, auxin, and methyl jasmonate, suggest potential roles in hormone signaling. Notably, ethylene-responsive elements (EREs) were found in *CmALKBH6*, *CmALKBH7*, and *CmALKBH8* promoters, indicating potential roles in ethylene-mediated ripening (Figure 5).

### 2.7. Impact of 1-MCP Treatment on Melon ALKBH Gene Expression

**Figure 6:**
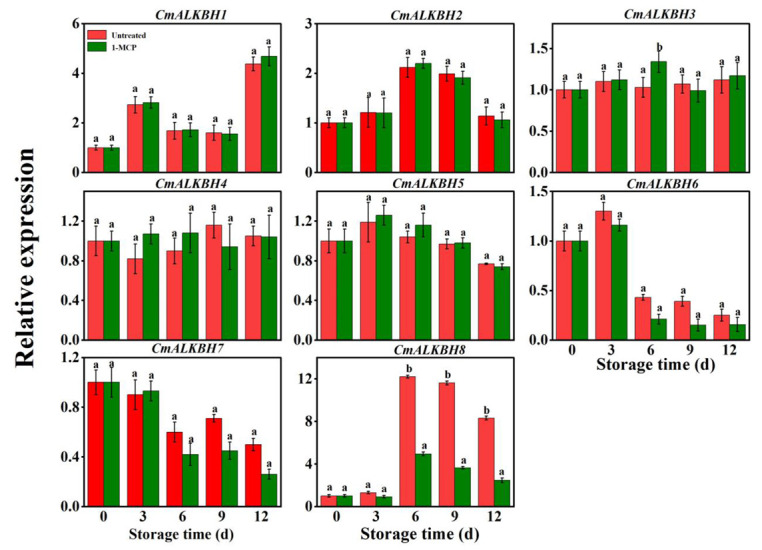
Effects of the 1-MCP treatment on the expression of the CmALKBH gene family during melon fruit storage. Values are shown as the means ± SE obtained from three independent experiments, and different lowercase letter above columns indicate significant differences (*p* ≤ 0.05).

To investigate whether CmALKBH genes are involved in ethylene-mediated ripening, we analyzed eight *CmALKBH* gene expressions via qPCR after treating oriental melon fruits with the ethylene inhibitor 1-methylcyclopropene (1-MCP). During fruit ripening, the expression of *CmALKBH1*, *CmALKBH2*, and *CmALKBH8* increased, whereas that of *CmALKBH3* and *CmALKBH4* showed no significant changes (Figure 6). The expression of other members decreased in later ripening stages. The expression of *CmALKBH6*, *CmALKBH7*, and *CmALKBH8* was inhibited by 1-MCP, whereas that of the other five genes was not significantly affected. This suggests that ethylene-regulated *CmALKBH* members may play a role in ethylene-mediated ripening.

### 2.8. Functional Exploration of CmALKBH8 in Melon Fruit Ripening

**Figure 7:**
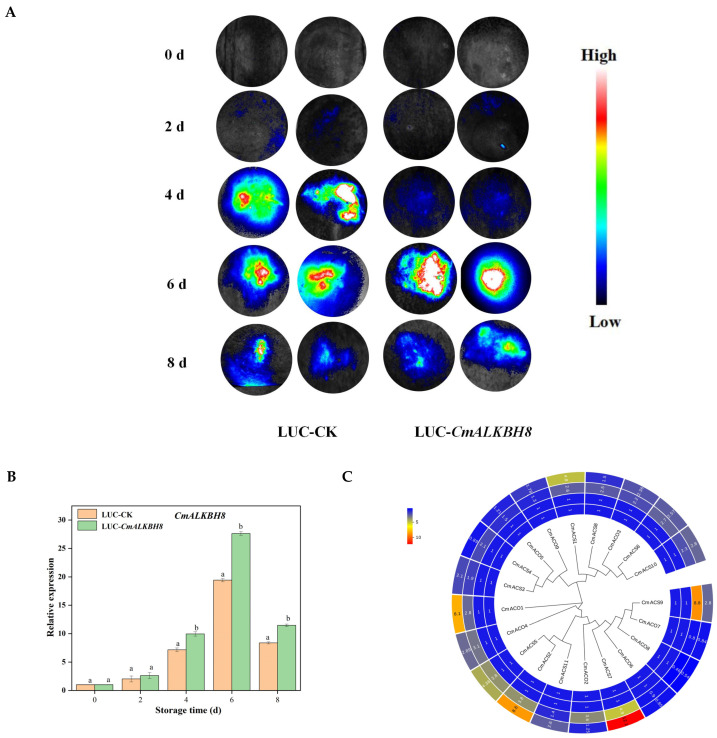
LUC fluorescence signal detection of transient overexpression of CmALKBH8 in oriental fruit and expression analysis. (**A**) Photos of fruit fluorescence signals in different days after agrobacterium-mediated transient injection. (**B**) Expression levels of CmALKBH8 in overexpression and the control fruit. (**C**) Expression levels of CmACS in CmACO family gene expression heatmap in overexpression and the control fruit. From inside to outside, the heatmap represents the control fruit at 0 d after injection, the overexpression fruit at 0 d after injection, the control fruit at 6 d after injection, and the overexpression fruit at 6 d after injection. Values are shown as the means ± SE obtained from three independent experiments, and different lowercase letter above columns indicate significant differences (*p* ≤ 0.05).

*CmALKBH8* expression was positively correlated with endogenous ethylene production during post-harvest ripening and significantly inhibited by 1-MCP. Promoter analysis revealed EREs, making *CmALKBH8* a candidate gene for functional validation. Transient OE in oriental melon fruits using Agrobacterium-mediated transformation showed increased luciferase fluorescence 2–6 days post injection, with slight weakening at eight days, confirming the successful transformation (Figure 7A). qPCR analysis confirmed the significant OE of *CmALKBH8* 4–8 days post injection (Figure 7B). the OE of *CmALKBH8* significantly raised the expression of ethylene synthesis genes, *CmACO1*, *CmACS1*, *CmACS2*, and *CmACS7*, whereas *CmACS9* expression was significantly inhibited; no other members showed significant differences. These results suggest that *CmALKBH8* may regulate ethylene synthesis by altering the expression of these key genes, thereby influencing melon fruit ripening.

## 3. Discussion

RNA m^6^A methylation is a widely distributed and highly conserved modification with crucial roles in eukaryotic RNA export, splicing, translation, and stability that has implications for fruit development and ripening [32]. However, its role in melon fruit ripening has not been reported. Therefore, we preliminarily explored the relationship between m^6^A modification and melon fruit ripening. MA, an m^6^A eraser inhibitor, increases m^6^A methylation levels by inhibiting demethylases (FTO or ALKBH5 homologs) [33,34]. Research on applying MA in fruit development and ripening tomato plants indicated that injections delayed ripening [31]. Therefore, we explored the efficacy of MA in oriental melon by employing the same injection method [31]. MA treatment 25 days after pollination delayed ripening, significantly reduced endogenous ethylene production, and extended the ethylene release peak (Figure 1). Similarly to climacteric fruits such as kiwifruit and tomato [10,23,32], our results suggest that m^6^A methylation may also regulate ripening in climacteric melon fruits. MA suppressed the expression of the melon m^6^A demethylase *CmALKBH* genes (Figure S1) and delayed fruit ripening.

The *ALKBH* gene family, as demethylases, participates in plant growth, development, and senescence, including fruit ripening. The completion of cucurbit genome sequencing provides detailed information for studying the melon *ALKBH* gene family. We predicted eight *CmALKBH*s to be demethylase genes, all containing the 2OG-FeII-Oxy domain. Many plants have undergone the complete genome-wide identification of *ALKBH* genes, with cotton having the most members (26) [35], poplar having 23 [36], and *Arabidopsis* [37], rice [37], tea [38], sugar beet [21], and litchi [39] having 10–16 members. In this study, we identified fewer members because of stricter HMM criteria, which was consistent with tomato [13], watermelon, and cucumber, indicating a lack of expansion in the ALKBH family during evolution. CmALKBH proteins vary in amino acid length, type, isoelectric point, and molecular weight (Table 1). A phylogenetic tree of ALKBH proteins from melon, watermelon, cucumber, *Arabidopsis*, and tomato indicated five subfamilies, with an uneven distribution indicating diversity (Figure 2). Evolutionary relationships showed that CmALKBH is closer to CsaALKBH and ClaALKBH than to AtALKBH and SlALKBH, which is consistent with synteny analysis (Figure 3). Many *Arabidopsis* ALKBH genes have confirmed functions, such as *AtALKBH9B*, which regulates alfalfa virus infection [40,41,42] and is syntenic with *CmALKBH1*, suggesting similar functions. Therefore, while CmALKBH1 and CmALKBH2 are located in close proximity on chromosome 8, this physical proximity does not imply functional similarity as they belong to distinct ALKBH subfamilies (Figure 2) and their expression patterns during fruit ripening differ significantly. All these divergences suggest potential functional differentiation. As their amino acid sequence identity was only 13.8% (Table S4), the observed discrepancies might also result from the incomplete protein sequence of CmALKBH2 obtained from the melon genome.

Promoters, located upstream of genes, contain various cis-acting elements that regulate gene expression throughout the plant lifecycle [43]. EREs mediate ethylene signaling and regulate gene expression, often located in promoter regions [44]. Ethylene, an important plant hormone, regulates downstream gene expression through EREs, affecting processes such as fruit ripening [45]. Melon ALKBH gene promoters contain diverse hormone-responsive elements (Figure 5). *CmALKBH6*, *CmALKBH7*, and *CmALKBH8* contain EREs, and their expression is significantly inhibited by 1-MCP, suggesting that ethylene may regulate their expression through EREs and specific transcription factors such as ERF or EIL, thereby influencing m^6^A methylation levels.

Growing research on the functions of *ALKBH* genes in plants has shown that different members regulate flowering, growth, development, and stress responses [19,20,34]. However, their roles in fruit ripening have only been reported in tomato [23]. Research using wild-type, *SlALKBH1*-OE, and *SlALKBH1*-knockout lines found that *SlALKBH1* increases the transcription of lycopene and carotenoid synthesis genes, ethylene signaling pathway regulators, and cell-wall degradation genes, enhancing ethylene sensitivity and promoting ethylene synthesis gene expression, leading to early ripening [13,22]. *SlALKBH2*, an endoplasmic reticulum-localized m^6^A RNA demethylase, directly targets and regulates the stability of the demethylase gene *SlDML2*. *SlALKBH2* mutation reduces *SlDML2* mRNA abundance, delaying ripening [23]. In this study, we also transiently overexpressed ethylene-regulated *CmALKBH8* in melon fruits. qPCR analysis showed significant increases in the ethylene synthesis genes *CmACO1*, *CmACS1*, *CmACS2*, and *CmACS7* (Figure 7), indicating that *CmALKBH8* may regulate ethylene biosynthesis by altering the transcription of these genes, thereby influencing melon fruit ripening. CmALKBH8 belongs to the AlkB family of demethylases, and we speculate that its effect on the transcriptional levels of *CmACO1*, *CmACS1*, *CmACS2*, and *CmACS7* is achieved by reducing the methylation levels of their promoters. Furthermore, CmACS9 expression was significantly inhibited, this might be related to the elevated expression level of CmACS1, which indirectly affects the expression of its homologous proteins. Alternatively, it could be due to the overexpression of *CmALKBH8*, which directly induces an increase in other types of methylation in the promoter region of *CmACS9*. However, further studies are still needed to establish a stable gene knockout system to verify whether the transcription and translation of *these genes* are altered after *CmALKBH8* knockout, whether the key steps of ethylene synthesis are slowed down, and ultimately whether this delays the ripening of oriental melon fruits.

## 4. Materials and Methods

### 4.1. Plant Materials and Treatment

The experimental material was the oriental melon ‘Xiaomifeng’, planted in 2024 at the Shenyang Normal University Life Science College Organic Vegetable Innovation Technology Research Base. Melon injection was performed based on previous melon injection methods [11] with some modifications. A 0.5 mm diameter syringe was used to inject 1 mL of 100 mM MA into ‘Xiaomifeng’ fruits 25 days after pollination. The needle was inserted 3–4 mm into the fruit pedicel, then 500 μL of 100 mM MA was injected until the fruit surface was fully permeated and droplets emerged from the calyx tip. Water injections served as a control. Fruit samples were harvested at 0, 3, 6, 9, and 12 days post injection. The experiment was conducted with three biological replicates, each consisting of six fruits. We have clarified this methodological detail in Section 4 of the revised manuscript.

Fruits were harvested 25 days after pollination and treated with 1 mg/L 1-MCP (0.14% 1-MCP powder EthylBloc^®^, provided by Rohm and Haas Company, Philadelphia, PA, USA) at room temperature, with water-treated fruits serving as the controls. Treated and control fruits were placed in 0.10 mm thick non-toxic PVC-lined boxes for 24 h. Sampling was conducted at 0, 3, 6, 9, and 12 days to measure the firmness, soluble solids content, and endogenous ethylene production. Fruit firmness measurements were conducted using an FHM-1 fruit firmness tester (Shenfen Instrument, Shenzhen, China) (12 mm base diameter) at the fruit midsection (with peel). Soluble solids were measured using a handheld refractometer (Shenfen Instrument, China). Ethylene was extracted from the fruit cavity and measured by use of a Varian GC-3800 gas chroma to graph (Varian, Palo Alto, CA, USA), with three replicates, under the following analysis conditions: GDX-102 column (Varian, USA), FID detector, non-split injection (Varian, USA), nitrogen carrier gas, column temperature 100 °C, FID detector temperature 120 °C, and flow rate 20 mL/min [29].

### 4.2. Identification of ALKBH Genes Within the Melon Genome

Based on previously released plant ALKBH protein sequences (accession number PF13532) [36], the HMM was downloaded from Pfam (http://pfam.xfam.org/) [46]. DNA, CDS sequences, and gene annotation files (gff3) for melon, cucumber, and watermelon were downloaded from the cucurbit genome database. HMMER 3.0 software was used with the ALKBH domain from Pfam as the search basis, setting a threshold of e < 1 × 10^−5^, to screen ALKBH proteins in melon, cucumber, and watermelon. ExPASy software (https://web.expasy.org/protparam/) was used to calculate protein physicochemical properties, such as the theoretical isoelectric point, molecular weight, and length [47]. ALKBH gene subcellular localization was predicted using the Cell-PLoc 2.0 server (http://www.csbio.sjtu.edu.cn/bioinf/Cell-PLoc-2/) [48].

### 4.3. Phylogenetic Analysis, Gene Structure, Protein Motifs, and Syntenic Analysis

We performed multiple sequence alignments of melon, watermelon, and cucumber ALKBH proteins with *Arabidopsis* and tomato ALKBH proteins using the ClustalX 2.0 software with default parameters [49]. MEGA 11 software was used to construct a phylogenetic tree using the neighbor-joining method with 1000 bootstrap replicates [50].

Melon CmALKBH gene CDS and gene sequences were analyzed for exons and introns, then displayed by use of the Gene Structure Display Server (http://gsds.gao-lab.org/) [51]. MEME v5.4.1 (https://memesuite.org/meme/doc/meme.html) was used to identify conserved motifs in CmALKBH proteins, with default parameters. CFVisual_2.1 was used for visualization [52].

MCScanX (2022) software was used to detect gene synteny [53], including CmALKBH genes with *Arabidopsis*, tomato, cucumber, and watermelon ALKBH genes. Circos v0.67 was used for synteny visualization [54].

### 4.4. Cis-Acting Elements Analysis of Promoter Regions and Gene Expression

Each upstream genomic DNA sequence of 2.0 kb length from melon CmALKBH gene was acquired from the cucurbit genome database. A search of potential cis-acting elements within promoter regions occurred through the utilization of the PlantCARE database (http://bioinformatics.psb.ugent.be/webtools/plantcare/html/) [55]. R studio 4.4.0 was used for promoter heatmap visualization.

Fruit RNA was extracted by use of the Tiangen (Beijing, China) RNAprep Pure Plant Total RNA Extraction Kit (Tiangen). The FastKing one-step reverse transcription-fluorescence quantitative kit (Tiangen) was used for qPCR, with primers designed using Primer 3.0 online. Real-time PCR was performed for gene expression studies according to the method described by our previous study [56]. Table S3 presents the primer sequences used for this study.

### 4.5. Transient Expression of Oriental Melon Fruit

Based on the melon genome CmALKBH8 sequence (MELO3C015210), cloning primers were designed using Primer3.0 (F: 5′-ATGTTTTTCATCCGTACACT-3′, R: 5′-TTAATACTTTCTAAAGGTAA-3′) to clone the CmALKBH8 CDS sequence. The CmALKBH8-pCAMBIA3301-LUC recombinant plasmid was constructed and transformed into Agrobacterium EAH105-competent cells. The pCAMBIA3301-LUC strain (negative control) was injected into fruits 25 days after pollination, with fruits harvested at 0, 2, 4, 6, and 8 days post injection [57].

### 4.6. Statistical Analysis

SPSS 18.0 software was utilized for one-way ANOVA. Excel 2019 software was used for graphs.

## 5. Conclusions

This study is the first to demonstrate that m^6^A RNA demethylase inhibitors delay oriental melon fruit ripening. Based on the cucurbit genome database, we comprehensively studied the m^6^A demethylase *ALKBH* gene family in melon for the first time. Eight *ALKBH* genes were identified, and their chromosomal distribution, gene duplication, and evolutionary selection were systematically analyzed. By determining the expression patterns of melon *ALKBH* genes during post-harvest ripening, we identified three ethylene-regulated *ALKBH* gene family members. A preliminary exploration of the function of *CmALKBH8* in melon fruit ripening showed that *CmALKBH8* upregulates the transcription of key ethylene synthesis genes, potentially accelerating ripening in fruits overexpressing this gene. This study elucidates the regulatory mechanisms of m^6^A RNA methylation and ethylene in oriental melon fruit ripening.

## Figures and Tables

**Table 1:** Physicochemical properties of ALKBH gene family members in *Cucumis melo*.

Name	ID	Protein Length (bp)	CD Length (aa)	Molecular Weight (kDa)	Theoretical pI	Chromosome Location	Predicted Location
CmALKBH1	MELO3C007897.2	500	1503	55.85	6.16	chr08:6086321 … 6092751 (+)	Nucleus
CmALKBH2	MELO3C008006.2	244	735	28.06	9.3	chr08:6722153 … 672334 (−)	Nucleus
CmALKBH3	MELO3C010538.2	342	1029	38.52	6.83	chr07:7974500 … 7982220 (−)	Chloroplast
CmALKBH4	MELO3C011863.2	363	1092	41.21	6.18	chr10:4296265 … 4298586 (−)	Cytoplasm
CmALKBH5	MELO3C017969.2	260	783	29.54	4.68	chr07:28389212 … 28391906 (+)	Nucleus
CmALKBH6	MELO3C024592.2	250	753	28.45	7.27	chr08:9071712 … 9074429 (+)	Exracellular
CmALKBH7	MELO3C025799.2	502	1509	54.91	9.01	chr04:18571821 … 18576784 (−)	Nucleus
CmALKBH8	MELO3C026046.2	472	1419	53.44	9.32	chr08:28592781 … 28595439 (+)	Chloroplast

## Data Availability

Data is contained within the article and Supplementary Materials.

## Supplementary Materials

The following supporting information can be downloaded at: https://www.mdpi.com/article/10.3390/ijms26094254/s1.
