# Supplementary material for: Identification and Functional Exploration of the ALKBH Gene Family in Oriental Melon Fruit Ripening
Source: Int J Mol Sci. 2025 Apr 29;26(9):4254. doi: 10.3390/ijms26094254 (PMC12071693; doi:10.3390/ijms26094254)
Supplement: Supplementary file 1 [file ijms-26-04254-s001.zip › ijms-3525415-supplementary.pdf]

Table S1. Conserved domain analysis of CmALKBH proteins

Gene	Conserved domain
CmALKBH1	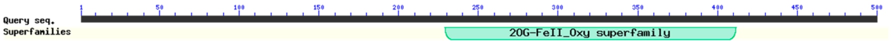
CmALKBH2	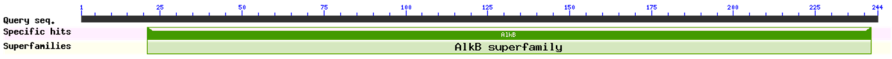
CmALKBH3	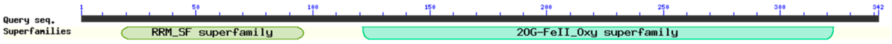
CmALKBH4	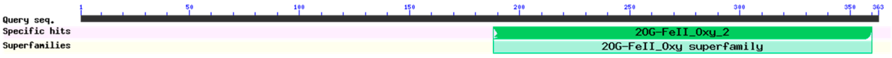
CmALKBH5	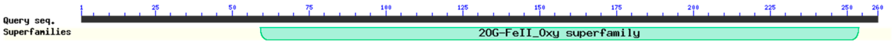
CmALKBH6	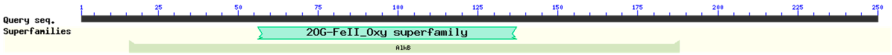
CmALKBH7	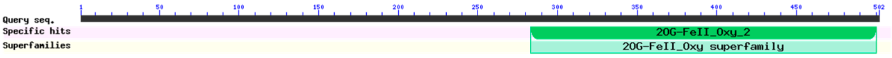
CmALKBH8	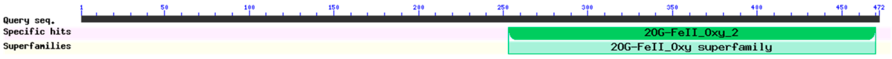

Table S2. Conserved motifs identified from the ALKBH genes in melon

Motif	Sequence
Motif-1	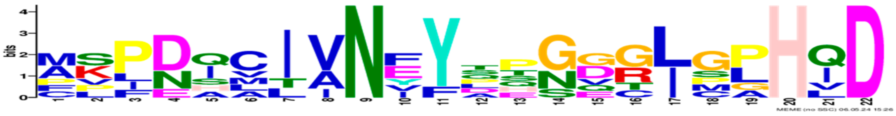
Motif-2	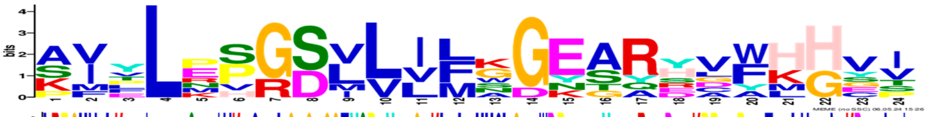
Motif-3	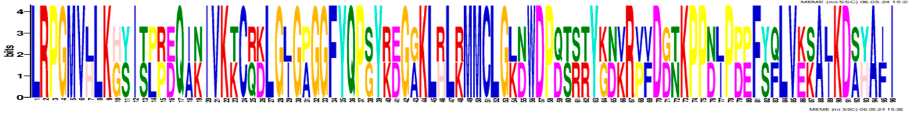
Motif-4	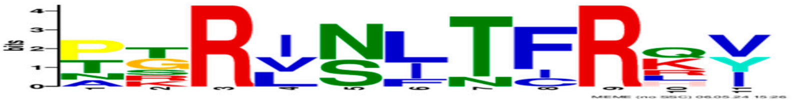
Motif-5	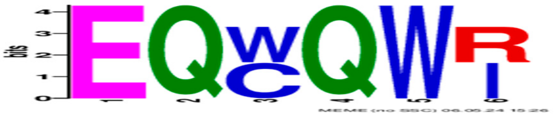
Motif-6	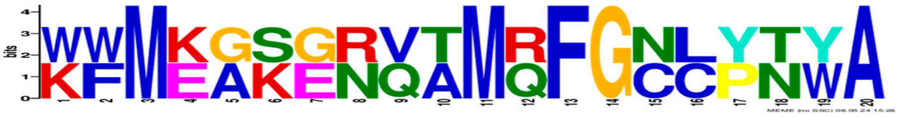
Motif-7	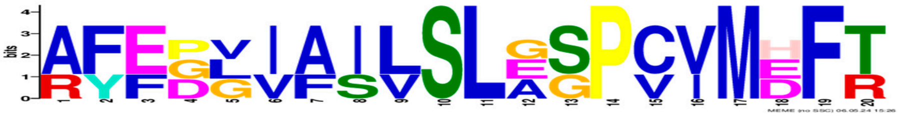
Motif-8	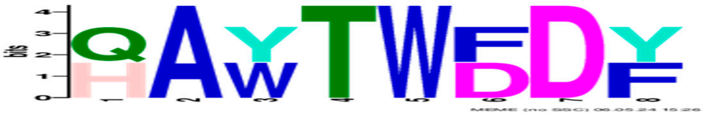
Motif-9	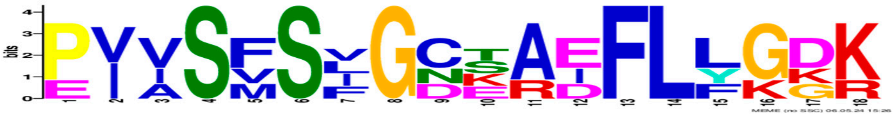
Motif-10	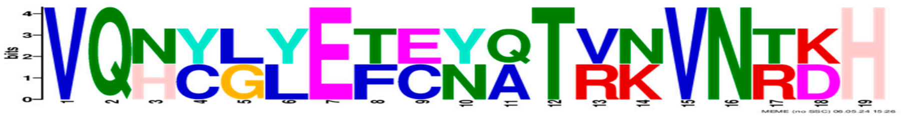

Table S3 Sequence of primers

Accession number	Size of amplified (bp)	Sequences(5′ to 3′)
MELO3C021182	182	F-CCTCCTCCTCCAAGACGATC<br>R-CTGCTACCGTGTTTCTCAGC
MELO3C016340	118	F-GTGACCTCTGCGATCGAAAC<br>R- TCTACCCCAGTTCGCCATTT
MELO3C019008	214	F- ATCACCATTGCCCAAACCAC<br>R-GACTAAGCCTTTAACGCCGG
MELO3C007662	225	F-AACGCAAAGACACCATCGAC<br>R- TTGGTGATGGCTGCAGAGTA
MELO3C021182	182	F-CCTCCTCCTCCAAGACGATC<br>R-CTGCTACCGTGTTTCTCAGC
MELO3C016340	118	F-GTGACCTCTGCGATCGAAAC<br>R-TCTACCCCAGTTCGCCATTT
MELO3C015444	166	F-GCTTGTCCGAGAACAGGTTG<br>R-AAACCTGCCATCGCCATTTT
MELO3C005597	143	F-GGTTGATGTCCCTCCAATGC<br>R-TGTTTCCATCCTCCTGTGCT
MELO3C010779	128	F-ACGTTTCCACTACCGCTACA<br>R-GTTCATAGTGGAGCTTGCGG
MELO3C006840	118	F-TTGAAGGATGGGCAGTGGAT<br>R-AAACCCGATGCCAAACACTC
MELO3C024891	210	F-GGAGCAAGCCCAAACATTGA<br>R-TGTAGGTTCGGGTCCATGAC
MELO3C024892	158	F-TCTTGGGTTGGAGAAGGGTT<br>R-GGAAGAGGAGGATGATGCCA
MELO3C024893	269	F- CTCTCCAAAGATCTCGGCCT<br>R-AACAAACCGGCATTCCCTTC
MELO3C014437	273	F-CAGAGCCCGGTTTCATTAGC

		R-CAGCACTCCCAGCAAGAAAG
MELO3C004619	274	F-GCAGTACCTTTTGTTGGCGA
		R-CAATGGCATGACTGTCCGAG
MELO3C007425	182	F-CGCCGATCTCCCAGAACTAT
		R-TGCTGACTTTTGTGCCGAAA
MELO3C006438	107	F-CGCCAAAGAAGAAGCATCGA
		R-AGGTGACATCATGTGGGGAG
MELO3C006439	142	F-GGGAGTAAAAGGTTTGGCGG
		R-ATTCTTGGCCTCTGTTTCGC
MELO3C019735	102	F-TTCGCTACTACCAACGCCAT
		R-TTTGTGAGTTCGCGTCCATG
MELO3C026436	113	F-CGACATGAACAAACTGGTGC
		R-CTGGATAGTAAGGAGTGGGAAC
MELO3C022978	150	F-AAAGCTAGCCATGATTCTCGT
		R-ATTTGGATAATGCCGGTGGG
MELO3C010508	273	F- AGGAATTGCTGTGTGAGGGA
		R-CGATCTGAAGCAAGTCACCG
MELO3C007897	239	F-TTTAGCTTCATTCCCTCCGTTCA
		R-CCAAGGATCTCTGCGGA
MELO3C008006	224	F-GTTGCACATCCTGGATCGAC
		R-GCAATCTCCTGGTTCACTGC
MELO3C010538	189	F-GAATCAGCCACCCCACAATC
		R-AACGTATGTGCAAAGTCCGG
MELO3C011863	216	F-GATCAGATCGCATGTAGTCGCCTTC
		R-GACAAAGGCACCGAC
MELO3C017969	192	F-CCACAAGACGGATACCTCGA
		R-TCGAGGGAGACAATGGCAAT
MELO3C024592	201	F-AGAAGGCTGCAAAATTGGGG
		R-AGGAAAGTAAGCAGGGCCAT
MELO3C025799	155	F-AGGTGAGGGGTCTTCTGTTG

		R-TATTTAAAGGCCGCCACTGC
MELO3C026046	214	F-GTTACGGACCATTCGCTTCC<br>R-GCCCAAGACCAAGCTTTTGA
	148	F-AAACGGCTACCACATCCA<br>R-CACCAGACTTGCCCTCCA

Table S4 Homologous percentage of ALKBH amino acid sequences in melon(%)

CmALKBH	CmALKBH1	CmALKBH2	CmALKBH3	CmALKBH4	CmALKBH5	CmALKBH6	CmALKBH7	CmALKBH8
CmALKBH1	100							
CmALKBH2	13.8	100						
CmALKBH3	16	17.1	100					
CmALKBH4	15.2	21.3	19.2	100				
CmALKBH5	16.2	17.4	33.06	30.9	100			
CmALKBH6	13.2	14.8	25.93	14.2	18.5	100		
CmALKBH7	16.9	18.9	19	31.45	16.8	18	100	
CmALKBH8	16.6	21.9	19.9	28.95	17.5	14.6	50.64	100

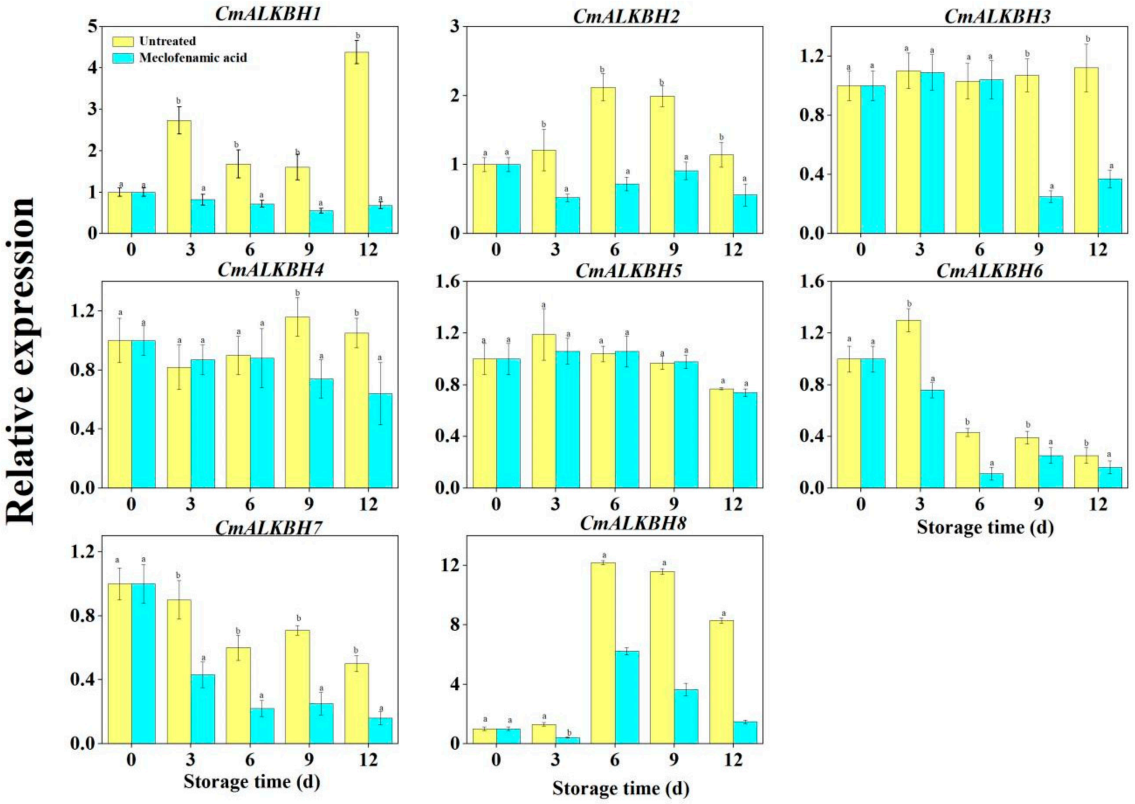

Figure S1. Effects of direct injection of Meclofenamic acid on oriental mleon *CmALKBH* gene expressions.
